# The HURT (Headache Under-Response to Treatment) questionnaire: utility in a specialist care center in Denmark

**DOI:** 10.1186/1129-2377-14-S1-P97

**Published:** 2013-02-21

**Authors:** ML Westergaard, RH Jensen, TJ Steiner

**Affiliations:** 1 Danish Headache Center, Denmark; 2 Imperial College London, UK; 3 Norwegian University of Science and Technology, Norway

## Background

The HURT Questionnaire was developed by Lifting The Burden, a non-governmental organization working in official relations with the World Health Organization, as a tool to aid in the management of headache. It has eight questions which the patient answers as a measure of effectiveness of intervention, by indicating when outcome is less than optimal, and by suggesting what changes in management might lead to improvement.

## Objective

The objectives of the study were a) to assess test-retest reliability of HURT and b) to show responsiveness to treatment-induced change.

## Methods

The questionnaire was administered on three occasions in a specialist headache center: pre-visit, at first visit, and when the specialist judged that the best possible outcome had been achieved.

## Results

Of 143 patients, 114 completed all questionnaires and records of 110 were available for analysis. They were mostly female (2:1), with mean age 44.4 years and headache duration of 15.6 years. Internal consistency reliability was ą=0.79 to 0.90. Test-retest reliability varied widely: highest for the question on number of days of headache per month (r_s_=0.84, kappa=0.68) and lowest on delaying medication because of side effects (r_s_=0.33, kappa=0.27). Responses post-intervention compared with baseline indicated a favourable outcome overall (80% of patients), reflecting specialist assessment that the best possible outcome had been achieved. There was no improvement in concerns about side-effects of medication (p=0.28). There was a significant association between improvement in HURT total score and migraine diagnosis (p=0.04). Patients with migraine showed the biggest changes in total scores. Records of non-responders were reviewed and there were no significant differences in terms of age (p=0.28) and gender (p=0.15), although 12 patients who were discharged after the study period were followed-up for a significantly longer time (p<0.001).

## Conclusions

The questionnaire can help patients describe headache symptoms, disability, medication use, self-efficacy, and knowledge about headache. It has utility in a specialist care setting but must be tested in primary care, for which it was originally designed.
